# Using Mobile Phone Apps to Deliver Rural General Practitioner Services: Critical Review Using the Walkthrough Method

**DOI:** 10.2196/30387

**Published:** 2022-01-25

**Authors:** Belinda O'Sullivan, Danielle Couch, Ishani Naik

**Affiliations:** 1 The Rural Clinical School, Faculty of Medicine University of Queensland Toowoomba Australia; 2 General Practice Supervisors Australia Bendigo Australia; 3 Monash University School of Rural Health Bendigo Australia; 4 Bendigo District Aboriginal Cooperative North Bendigo Australia; 5 University of Queensland Medical School Brisbane Australia

**Keywords:** rural health, mHealth, general practice, cell phone, rural health services, mobile applications, primary health care, morbidity, mobile phone

## Abstract

**Background:**

The widespread use of mobile phones represents new frontiers for improving access to health care. This includes using mobile apps to deliver general practitioner (GP) services in rural areas. However, the wider adoption of apps for increasing access to rural GP services relies on understanding how they might intersect with the rural health system context.

**Objective:**

This research aims to critically review mobile apps for delivering GP services in a rural health service context using the walkthrough method.

**Methods:**

The sample comprised 3 GP service apps under the top 100 list in the medical category in the Apple App Store (also available via the Google Play Store) in Australia as of June 2020. The walkthrough method was applied to extract data and critique the *explicit factors,* such as the app interface elements, and *implicit factors,* such as the embedded cultural features related to use for people in rural settings. Data analysis was undertaken between 3 researchers over 6 months applying the walkthrough method and using critical reflection.

**Results:**

There were 3 main themes: improving rural access, addressing rural health care needs, and providing quality of care. App-based GP services may improve rural GP service availability. However, this may be at a relatively superficial level that does not encompass the scope and intensity of the services needed in rural areas (including relevant chronic and emergency care) at a cost that rural patients can afford. The apps showed signs of limited tailoring to the cultural dimensions of rural health care as a barrier to rural use. Patients generally self-selected to use GP service apps with limited support, potentially leading to inappropriate uptake especially by disadvantaged groups with lower health literacy. Although the apps claimed to avail most GP services (70%-80% in some cases), it emerged after enrollment that emergency, complex, and serious conditions might be excluded, potentially imposing more complex caseloads on in-person rural GPs. Apps provided limited information about continuity and coordination of care and sharing information with rural GPs, potentially leading to fragmented and low-quality care. There was commonly no assurance of rural skills and experience of physicians staffing apps despite the wider scope of skills needed to be effective in rural general practice.

**Conclusions:**

GP apps may increase the availability of GP services, but they may require clearer exclusions, appropriate use through decision-making tools, more rural-tailored interfaces, and capacity to align appointment times and costs with patients with complex needs to engage and be useful in a rural context. It is also important to consider how these app-based services could share information with local health care staff for safety and continuity of rural primary care. Finally, information about the physicians’ rural training and experience is critical for quality.

## Introduction

### Background

Although health is one of the fundamental rights of every human being, poor access to health care in rural areas remains a major global issue impeding equity. At least half of the world’s population lacks full coverage of essential health services such as primary care [1]. This situation worsens in countries with a more rural population distribution. Where rural populations exceed 70%, only 16% have universal access to services [2]. The World Health Organization has a range of global strategies to increase access to rural health care, including developing the rural health workforce [3] and improving health care affordability [4]. Increasingly, mobile (cellular) phones are being considered a new frontier for improving universal access to rural primary care services as part of mobile health (using mobile and wireless technologies) [5]. This includes delivering general practitioner (GP or family physician) services via mobile phone apps to give rural people access to timely primary care by skilled physicians regardless of where they live and where the physicians are distributed [5]. Governments may be attracted to this because this strategy has the potential to be cost-effective and to enable real-time responsiveness to rural needs. However, widespread adoption depends on evaluating whether GP service apps can achieve the same goals as in-person GP care and not widen rural disadvantage.

Much of the literature has focused on using mobile health in rural areas for selective functions, including enhancing referrals; improving access for target populations such as birthing women; and as an adjunct to other forms of care [6-9] supporting rural health workers [10-12], supporting self-management [13], and delivering health promotion interventions [14]. There is limited research on the use of apps for the delivery of holistic primary care by GPs, which typically involves an array of first point of contact screening, diagnostic, intervention, and referral services that most of the population needs [15]. This is an important area to understand if GP service apps are to be adopted as a potential alternative to in-person GP service models. GP service apps may play a role in rural communities that have no GPs or too few GPs for the level of demand. GP service apps may offer convenience to rural patients as well as lower costs compared with the time, travel, and consultation fees they may face for in-person GP visits, although this has not been appraised.

The context of rural health care provides an important backdrop for critiquing apps. A major international agenda is to protect the health of the rural poor by availing health care that is needed (at the depth of coverage and intensity required) and in such a way that nobody suffers financial hardship as a result of obtaining the services they need [16]. This is challenging as rural populations have relatively more acute and chronic health care needs in low-, middle-, [2] and high-income countries [17], which increase with remoteness from urban centers. Beyond regional centers, towns of <50,000 population have access to fewer local health care providers (and other physicians), where rural GPs typically provide a broader range of both primary care and other specialist areas (approximately 10 additional hours in hospital atop of a typical primary care workload) [18,19]. They enable lifesaving procedural care for rural women and children and respond to medical emergencies [20,21], facing undifferentiated presentations that demand problem solving within limited resources [22]. In Australia, the achievement and maintenance of skilled GPs to service rural areas has been noted to require specific investment in rural-based training and ongoing professional support [21,23,24]. To this end, approximately 58% of Australia’s rural GPs engage in educating the next generation of GPs [25]. Introducing apps that could substitute for skilled rural generalist physicians and address the breadth of community needs may be challenging.

Evidence points to the need for rural GP service models tailored to population needs, including respecting community characteristics, cultural aspects of care, and the need for self-determination [26-28] to cater to the higher proportion of older adults, poor people, and First Nations people in many rural settings [29]. Adaptability and careful design underpin the viability of these models as contexts become increasingly remote [27,30,31]. It is unknown how well apps may be delivered within these dimensions or effectively reach more remote communities than in-person services can achieve.

Ensuring that rural GP care is affordable is inherent to enabling access by the rural poor. This may vary widely by health care insurance schemes in different countries. In countries such as Australia, a national Medicare policy provides a universal health insurance system to rebate the cost of GP care at the discretion of the GP [32]*.* In >80% of GP consultations, GPs *bulk bill* patients, resulting in no out-of-pocket costs [32]; however, this is less common in rural areas, where GPs tend to set their fees higher than the government rebate [33,34]. The degree of cost that apps may impose against the flexibility and ceiling of any patient charges for in-person care per country may be important to affordability.

The rapid growth of apps is recognized as having the potential to challenge issues of access, quality, and safety for different patient groups [35]. For rural populations, there may be gaps in digital inclusion, including for low-income rural populations, aging cohorts, and those with limited education and employment [36,37]. Some web-based platforms may segment the population, primarily targeting relatively healthy people in employment [38]. Although apps may allow for more client-centered health care, this also needs to be evaluated considering the findings of research that note that patients perceive safe and high-quality health care as bound to their interaction with a trusted physician [39]. This may depend on how well apps can accommodate a relationship with the same physician and the health service over time.

### Objective

With this background in mind, our research aims to critically review mobile apps for delivering GP services in a rural health service context using the walkthrough method.

## Methods

### Context of Study

Australia was chosen as a case study for this research given its extensive focus on delivering rural GP care for 29% of the population living rurally across a wide geographic landscape [40]. It is also invested in rapid policy development in digital health, proposed as a modern means of delivering safe, high quality, and effective health services [41]. In 2017, 80% of Australians owned a smartphone, and digital health care uptake increased during the COVID-19 pandemic, potentially laying the groundwork for increased use of digital health care delivery [42].

### Walkthrough Method

We used the walkthrough method as described by Light et al [43]. This is based on the Actor–Network Theory which foregrounds experience as shaped by sociocultural and technical processes. It provides a lens through which one can understand how user interfaces and functions within technology mediate social processes within a system of networks. Networks can include humans, things, ideas, or concepts [44]. This allows for an in-depth analysis of the complexity of apps and their aims to uncover traces of their inner workings, intentions, positioning, and environment of expected use. The walkthrough method involves active engagement with an app via step-by-step progression through the interface using an environmental and technical scan via a structured template (Multimedia Appendix 1) [43].

Although the walkthrough method has been used to critically review communication, media, and cultural apps, few studies have applied it to appraise health care delivery—1 study evaluated an app for mental health self-monitoring and another focused on disease surveillance and tracking [45,46]. For this reason, we first established the boundaries of the project by writing and agreeing on a study protocol, which included the intention to explore *explicit factors,* such as the interface elements themselves, and *implicit factors,* such as the embedded cultural values framed by these elements, and how these may intersect with the rural health system.

Within the technical walkthrough, we also applied the framework of person-centered access to health care within systems by Levesque et al [47]. This framework specifically addresses the opportunity for consumers to identify health care needs and seek, reach, obtain, and use these services to achieve their goals. This ensured that the technical walkthrough collected structured data specific to health care provision to critique issues around approachability, quality, relevance, and satisfaction.

### Sampling Frame and Data Collection

We sampled the 3 most used GP service apps that offered consultations from the Apple App Store under the top 100 list in the medical category as of June 2020 in Australia to ensure we critiqued apps with the highest levels of use. We sampled these apps as we wanted to understand a range of apps that people are likely to experience to access GP services. The chosen apps were independent services not affiliated with GP services that were based in a physical location. These apps were also available via the Google Play Store.

The researchers first downloaded each app (called apps 1, 2, and 3) and established a dummy profile. This was done by entering all the normal client information such as the name and payment information required by the apps to register as a client. This enabled the researchers to access all the app functions for a thorough analysis. None of the researchers had used apps to access GP services before commencing this research, and none were health care providers to minimize bias. In total, 2 researchers had iPhones and 1 had an Android device, allowing for a complete analysis that accounted for any difference between platforms. Over a 10-week period, the researchers used a preagreed walkthrough template (Multimedia Appendix 1) to independently explore and document reflections about the elements of the app and its background. This was done by each researcher exploring the app independently to promote critical analysis. They also read about each app on the related webpage and on the Google or Apple store as well as in any of its business reviews (material about the app available on the web). There was no interaction with patients on the web or with GPs as the purpose was to engage with the app interface. The environmental scan explored the context of expected use, including social, political, economic, and cultural contexts, and the technical scan explored mediator characteristics of actual use, with room for additional comments. Screenshots were also recorded to assist with data interpretation and discussion.

### Analysis

Each week, the researchers (BOS, DC, and IN) met and reflected on their independent data from field notes, screenshots, and emails received from the apps, with a researcher (IN) summarizing this discussion in written notes. From this discussion, several conceptual ideas arose, including the need to confirm or disconfirm theories or practicalities. This involved the researchers identifying and sharing relevant literature and revisiting the app to update new observations. Any emerging findings about an app were used to stimulate deeper exploration of the other apps. This was done until clear findings emerged that allowed for sufficient critique across the 3 apps with respect to the rural health system. The research team continually discussed arising material, critiquing the apps through sociological, patient, and system lenses and reading more widely about the rural health system to agree on the 3 cross-cutting themes that are presented.

Our research team included 3 researchers of mixed career stages skilled in clinical, public health, rural health services, and health sociology to aid reflexivity (testing biases and assumptions). One was employed in a rural First Nations community-controlled health service, another in a Rural Clinical School, and the third was employed as a city-based medical student. In total, 2 had experience working clinically in primary care (as physiotherapists). All were women.

This project did not require ethics as there was no data collection from humans or animals, and only publicly available data about a system of care, rather than patients, were used.

## Results

A total of 3 key themes were identified, which included how the apps related to improving rural access, addressing rural health care needs, and the provision of quality services.

### Improving Rural Access

The apps targeted rural access within a wider market offering of accessing GP services from anywhere (Multimedia Appendix 2). A business review (app 2) claimed that the service was appropriate for *“*patients in metro, regional, remote and foreign locations.*”* App 3 also claimed that they supplemented access for towns in a context where they had no GP *“*or if it does, they may be booked out for 3 or 4 weeks.” The apps were mostly founded by non-GP professionals (apps 1 and 3). App 1 worked as a stand-alone MedTech company (allowing for public trading and investors), and app 2 was an Australian Stock Exchange–listed company (which is ranked by market capitalization). This suggests that apps offer business opportunities for nonmedical entrepreneurs. Despite profit motivations, all the apps sought to position themselves as affordable as part of promoting access:

...consultation costs are extremely competitive and lower than any private practice for a standard consultation...

App 3

User costs were mostly hidden in the app’s background information (except in app 3, where users could choose to read frequently asked questions [FAQs], including information about costs, before booking; Multimedia Appendix 2). Otherwise, costs had to be deducted at the point of booking based on displayed appointments, each of which noted the price the user would pay (apps 1-2; Multimedia Appendix 2). When trying to book an appointment, it became apparent that app-based services were billed at fixed time slots (10-15 minutes) using fully private billings to patients (Aus $40-$60 [US $28.69-$43.04]; apps 1-2) without reimbursement from Medicare (selective Medicare allowances for app 3 and timed appointments ranging from Aus $35 [US $25.10] to Aus $105 [US $75.31] by time category; Multimedia Appendix 2). It was necessary to enter payment details before booking an appointment. This mainly required users to have a credit or debit card (app 2 had some other options for payment for people without a credit card; Multimedia Appendix 2). Although the apps offered appointment options that were promoted as “instant*”* and “on demand,” it was not explicit as to whether this related to accessing a consultation with any particular physician and being able to see the same physicians over recurrent consultations or whether this may vary widely between different occasions of use.

Visual imagery was extensively used within app 1 and on all the apps’ websites to support engagement. Predominantly White women were depicted, with perfect skin and painted nails, presenting as mildly unwell (indicated by a box of tissues or a thermometer) sitting in clean houses with quality furnishings and clean and up-to-date technology devices. The images emanated users having positive interactions with the GP provider on the web, smiling and waving at the screen implying a personable relationship that is at odds with the app’s ideal of real-time care of any GP available on the day. The images of physicians presented were also mostly White, elegantly groomed women or late middle-aged men in white laboratory coats with stethoscopes. However, when choosing an actual GP appointment in app 1, the image of the GPs with whom real appointments were made and the information about their country of training depicted providers of widely differing cultural backgrounds and overseas qualifications. In terms of approachability, the apps were differentiated by various opening hours and wider availability than in-person GP services (all offered GP care 7 days per week, one noted coverage 24/7, and app 1 allowed overseas clients; Multimedia Appendix 2).

The user’s technology requirements were relatively flexible (Multimedia Appendix 2) and, therefore, accommodating. However, there was an assumption that the user would have digital access and find the technology:

...easy to use our service...all you need...just click on the button...

App 1

### Addressing Rural Health Care Needs

The apps used marketing to position their utility as responsive, high-quality, simple, comfortable, and affordable health care regardless of location:

...patient-centred...convenient, quality healthcare...at a time and location that suits you...[enabling healthcare that is]...simple and affordable.

App 1

They also claimed that they could provide most GP services (on website promotions): “70% of GP presentations can be handled by telehealth (*)” (app 1), which they claimed was based on American research, but this was not cited. Furthermore, it was claimed that “80% of normal GP services can be done online” (app 2), with no justification for this (Multimedia Appendix 3). The apps did not collect critical information about the patient’s rural context, such as their town name; access to local hospitals, pharmacies, or specialists; distances from referral centers; and transport options, despite these factors being strongly tied to the capacity to address rural health care needs.

The apps proceeded to claim that they delivered a wide service range: *“*we do everything*”* (app 1). However, their booking features (Multimedia Appendix 3) depicted more limited allowances, covering prescriptions, medical certificates, referrals, or other (seeing a GP for something more general). With reference to apps 2 and 3, the nature of the services available was only described under the *FAQs* and *learn more* sections that patients could choose to view or not before booking; however, these sections were not obvious to the user. There was also a conflicting narrative about the range of services that the apps offered between what was described on the app’s website and the app itself. The app 2 website denoted use for weight loss, alcohol, and drugs, although this utility was not mentioned in the *learn more* section of the app.

Using the app for *other* functions (app 3) suggested poorly defined boundaries. There was no warning of exclusions or support to help patients discern the relevance of the service for addressing these needs, including any exclusions for a physical examination or for presentations such as pregnancy. Mostly, the apps provided refunds if users cancelled appointments—app 2 did so only if cancellations were given with 24 hours’ notice, thereby excluding the app’s purpose of accessing immediate care, and app 3 did so if the GP deemed the patient unsuitable (Multimedia Appendix 2).

It emerged that, despite their claimed utility, all apps excluded emergency services (only noted via the websites of apps 1-2; Multimedia Appendix 3):

If you have a medical emergency please stop now and contact local emergency services.

App 2

Only app 3 included FAQs to define emergencies:

...chest pain, head or spinal injuries, severe bleeding, loss of movement, breathing difficulties and reduced level of consciousness.

App 3

This definition provided clear boundaries but excluded a range of early warning symptoms such as headaches, sensory changes, or loss of balance that could be equally considered emergency situations in rural contexts.

Complex care was also excluded (Multimedia Appendix 3):

Doctors reserve the right not to treat you if you are complex.

App 1

This service is not suitable for serious medical conditions which should be handled by your GP.

App 1

Complex care was not defined, and there were no qualifiers as to what conditions might be *“*serious,*”* including no material on the website’s FAQs to guide decision-making. It became apparent for app 1 that this exclusion was related to the time involved in managing such cases rather than the limitations of the web-based model:

Complex medical problems may not be suitable for Telehealth Consultation as they may require a longer time than 15 minutes.

App 1

Additional service exclusions were evident when attempting to book a consultation with an individual physician on apps 2 and 3. Further exclusions were noted for lengthy issues requiring physical examination or difficult negotiations:

[We are] unable to process mental healthcare plans.

Apps 2 and 3

Fit for Work Certificate, Centrelink Certificate...you will need to present to a GP in-person.

App 3

...schedule 4 drugs that have the potential to cause harm should be sourced from your regular GP.

App 1

### Providing Quality Services

App 1 employed GPs who were generally registered but not qualified through the Australian General Practice training colleges—The Royal Australian College of General Practitioners or the Australian College of Rural and Remote Medicine (each requiring 3-4 years of vocational training and rural-specific training to prepare physicians for working in rural settings). The apps provided information about the title of qualifications the physicians held and the country the qualifications were obtained in. Apps 2 and 3 used Australian-qualified GPs; however, for app 3, it was not possible to see the qualifications of the GPs until the user’s payment details were entered (Multimedia Appendix 4).

For the GPs employed in app-based services, there was no information about rural skills or experience or about any cultural safety training that they may have done or not to ensure capability to provide quality rural GP care. For app 1, the GPs were subcontracted providers with their own Australian Business Number (Multimedia Appendix 4), and the app noted that the GPs’ advice was not guaranteed:

[We] do not represent, warrant or guarantee the quality of any medical advice provided by a doctor during a consultation.

App 1

Apps 2 and 3 did not disclose how the physicians were employed or make any disclaimers about the quality of their GPs’ services (Multimedia Appendix 3), whereby it can be assumed that their advice to rural and remote patients is guaranteed. None of the apps mentioned clinical backup for the GPs providing the consultations should an urgent situation arise or the physician need to advise on something beyond their scope of experience. In app 2, there was a disclaimer about the capacity of the GPs to deliver the services users may want under some circumstances, although the nature of the “circumstances” underpinning this situation was not clear:

Circumstances beyond our control may render it impossible to offer you an adequate service in which case you should seek the services of a local doctor.

App 2

The claims of safety of the app-based services were founded on the quality of the technology and guidelines around telehealth consultations rather than the quality of the medicine (Multimedia Appendix 4):

...using the latest in web technology...our health practitioners comply with all relevant professional standards including the RACGP Standards for telehealth and the Australian Health Practitioner Regulation Agency (AHPRA) Guidelines for technology-based patient consultations.

App 1

No apps mentioned how health care would be followed up or coordinated between providers or by the same GP if the app was used again (Multimedia Appendix 4). Only app 2 made provisions for patients to see their own physician if their own physician registered with the app-based service as a potential revenue generator for the app supply chain and its service volume.

App 2 also had a strong platform to promote web-based Medicare-rebatable specialist referrals; however, these were not guaranteed to occur in coordination with a rural user’s regular GP. The apps did not provide any information about teaching or professional support for the employed GPs. There was no training framework embedded for supporting workforce development, although this is a legitimate part of quality within any primary care ecosystem.

App 1 provided an option for script renewal without needing to see a GP:

...request a repeat prescription for a select range of medications they have used before by simply completing an online questionnaire...

App 1

Finally, all the apps only shared notes with the regular GP if the patients requested it (Multimedia Appendix 4).

## Discussion

### Improving Rural Access

Apps represent a major departure from traditional physician-led general practice businesses that have emerged from a professional philosophy within medicine of treating the ill to the best of one’s ability under ethical standards (conscience, integrity, and confidentiality), relegating livelihood to a secondary issue [48]. Their strong profit motivations may counter any mission to improve rural access and deliver the range of services needed by rural patients, particularly if doing so could be costly.

Although the apps made claims of affordability, they did not give explicit and up-front information to users about the costs they might incur in before engaging with the app, which could lead to unintended use and costs, deterring rural users from accessing ongoing primary care. The service structure around set appointment times and fixed payments may have limited benefits for improving access to services of the intensity, range, and affordability needed by rural people with chronic and complex care, including older adults and First Nations people who are overrepresented in rural communities [49]. Furthermore, it may be a barrier for rural users who may have lower education, higher unemployment, and volatile earning capacity (occupations susceptible to policy, business, and environmental conditions) [50]. In-person GPs can exercise discretion over appointment length and patient costs, including giving patients to the national Medicare rebate system, which flexes to consultation length and complexity at the discretion of the individual GP [33]. On the other hand, apps may not adequately address universal access goals of health care that aligns with need but is also affordable in a rural economy [16].

The apps are positioned in a way that counters rural community-centered norms, values, and culture, where health care is provided by physicians and health workers who are trusted and known in the community. First Nations people rely on services that are community-centered and that account for local community beliefs and values [49]. Instead, the apps seek to connect to an individual, shutting out the community context. What is offered is an aspirational lifestyle that is potentially at odds with the lived reality of the multicultural and rural poor. The reality of illness in rural overcrowded housing is juxtaposed against the elite and airy environment of the mildly unwell White woman presented [51]. Physicians depicted with stethoscopes seek to validate the provider’s skills as trustable, although the reality is that this claim cannot be tested by users through a web-based setting [52]. The different values portrayed may create uncertainty among rural users as to whether the app can work for them as promised, possibly hindering use. The lack of rural-specific language, culture, or dress of patients and providers in visual imagery could also deter rural and First Nations users from engaging with apps given that all aspects of health care need tailoring to promote access by groups that do not have Western medical ideals [49,53].

The focus on providing a payment mode before the consultation may challenge the rural psyche around a collective economy where goods and services are shared for community well-being and sustainability [54]. This rests within an Australian medical culture where GPs can be seen with no out-of-pocket costs because of the social values of fair and equitable health care [32]. Apps also somewhat oppose the rural ideals of self-determination and patient-centered care by positioning the protection of assets outside of the community as temporally more important than responding ethically and with ongoing commitment to supporting unwell people in a challenging environment.

Although the apps were marketed as easy to use, it is possible that older adults and digitally isolated people in rural and remote areas could find the use of apps challenging [37].

### Addressing Rural Health Care Needs

The degree to which apps account for 70%-80% of in-person rural GP services may need to be tested as to how well they align with the wider scope of rural GPs [18]. This relates to the role that in-person rural GPs play in addressing most medical needs in rural places where specialists and allied health providers are in shortage [55,56]. The assumption that apps can support most rural patient needs underplays the role of contextual and holistic patient- and place-based knowledge that is necessary for physicians to deliver effective GP services in rural places [22,57]. By espousing such a wide reach, apps are setting unrealistic expectations about how much they can assist rural users. One key example is that they do not disclose that they cannot provide physical examinations; however, Australia’s peak agency in web-based health, the Digital Health Agency, notes that telehealth is only useful “when a physical examination isn’t necessary” [58].

There is a clear orientation to profit over addressing rural needs as the apps were positioned to enable rural users to book inappropriate appointments because the service limitations were inadequately disclosed. A major policy review related to services provided by GPs recommended the use of clinical decision support tools to help with “the provision of advice at the point of care (when decisions are being made by the medical professional) that is tailored to the clinical context of the specific patient” [59]. Clinical decision support tools could be applied to promote the appropriate use of apps where indicated for rural patients.

Chronic and complex conditions are more prevalent in rural communities and, by excluding these conditions, apps are likely to miss the bulk of rural population needs. Some chronic conditions may be amenable to web-based consultation, for example, mental illness; however, the default referral of all complex problems to in-person GPs suggests that apps reject chronic illness to target a lucrative revenue base. Meanwhile, this may increase rural GP burnout and add to rural GP retention problems that already worsen with rurality [60]. This undermines the capacity of apps to address rural health care needs; rather, they have the potential to worsen rural GP burnout. Rural GPs seeing patients who have used app-based services may also need to spend more time chasing tests and referrals that patients may not easily recall. This may increase service duplication for patients and create inefficient and delayed care. The apps also failed to provide ongoing, coordinated care for rural people, which is inherent to supporting a high prevalence of chronic diseases in rural areas [61] and supporting aging and First Nations populations in rural communities [29].

With respect to delegating emergency care to local service providers, the apps assumed that rural users have access to such services locally. There was no attempt to screen for rural patients’ health service amenities, distance, and costs, as well as their emergency risks, when establishing appointments. The reality for many rural underserved populations facing distance and cost barriers is that complex health issues can remain dormant only to surface as an acute medical emergency at any time [22,62]. If apps do not screen for these issues appropriately and rural patients have no local GP to back up an app service, this could lead to emergencies being handled via app-based GPs unfamiliar with the service context and inefficient at mobilizing the local resources needed for lifesaving, rapid patient stabilization and retrieval [22].

Although apps propose a wide market position in GP care for rural populations, the Consumer Rights Law and the National Guidelines for Advertising of Regulated Health Services (section 29 of the National Law) suggest that apps may be marketing beyond their utility and creating unreasonable expectations of treatment. First, the Australia Consumer Rights Law requires that products do all the things that they propose they will do before people purchase services [63]. Second, the National Law requires that regulated health services do not create unreasonable expectations of beneficial treatment or encourage indiscriminate or unnecessary use [64]. The apps seem to nudge into the territory of doing both, particularly in light of their interface with rural consumers.

### Providing Quality Services

The major thrust of Australian policy aims to train and retain skilled rural GPs that can work effectively across the scope of patient care required in rural areas [21,23]. However, despite proposing to provide health care anywhere, the apps appointed GPs without disclosing their rural skills and experience and, in some cases, noted that they could not guarantee their advice. This contrasts strongly with the normal guarantees of in-person GP services, including the commitment of rural GPs to take on wider liability for patients where the caseload is vastly more undifferentiated and there are fewer health care resources [22].

The lack of support and upskilling provided for GPs working in the app business is unusual given the propensity for GPs to face difficult cases in rural areas. Countering this, rural health care quality is supported within in-person GP service models through team-based decision-making and clear escalation policies. The oversight of any service backup is perhaps managed by the app’s feature of self-selecting simple caseloads, but rural patients with complex or urgent care needs have a high chance of using the app inappropriately because they are inadequately screened and informed of exclusions before use. In addition, the apps did not mention anything about health worker training despite medical workforce supervision being an inherent part of rural GP practice quality improvement [65]. Training may be poorly accommodated within app-based models if it uses profitable time and has no payment attached to the learner.

There is some potential for app-based GP services to integrate better with local GP services rather than operating as stand-alone businesses. This could be led by rural GPs if a prototype app were developed for them to apply within their local business to manage waiting lists and promote early intervention. The benefits of this would be that the model occurs within the boundaries of an ongoing physician–patient relationship and reinforces rural GP business sustainability. Recent policy changes allowing in-person GPs to gain reimbursements via Medicare for new telehealth item numbers, incorporating rebates for services they deliver by telephone, support this expansion [66].

Although there was 1 example of an app proposing to provide for script renewal without an appointment, even small changes or continuation of medications may cause acute exacerbations in older adults and complex patients, which is common in rural settings. The app model depends on self-declaring existing medications, which may also be difficult for patients to recall. The result of this model may be that rural patients who have acute exacerbations will place pressure on limited rural staff and infrastructure atop of an already busy workload. Higher-quality care could be achieved if app-based consultation information were shared with a regular GP, thereby valuing rural physicians who understand the comprehensive patient history contextualized to place.

### Conclusions

In conclusion, app-based GP services may improve rural GP service availability. However, this may be at a relatively superficial level that does not encompass the scope and intensity of the services needed in rural areas (including relevant chronic and emergency care) at a cost that rural patients can afford. Apps show signs of limited tailoring to the cultural dimensions of rural health care, which presents a key barrier to rural use. Patients generally self-select to use apps with limited support, potentially leading to inappropriate uptake especially by rural cohorts who may be disadvantaged. Although apps claim to avail most GP services (70%-80% in some cases), after enrolling in these services, it emerges that emergency, complex, and serious conditions may be excluded, potentially imposing more complex patient caseloads on in-person rural GPs. They also provide limited information about continuity and coordination of care and sharing information with rural GPs as a source of fragmented and low-quality care for rural patients. There is commonly no assurance of rural skills and experience of app-based medical staff despite the wider scope of skills needed to be effective in rural general practice. It is advisable for app-based GP services to attend to these issues to better address rural access and health care needs.

## Appendix

Multimedia Appendix 1 Data collection template related to the walkthrough method.

Multimedia Appendix 2 A summary of the app business models and allowances.

Multimedia Appendix 3 A summary of the app appointment functions and utility.

Multimedia Appendix 4 A summary of the app quality, privacy, and troubleshooting.

## Abbreviations

FAQ: frequently asked question
GP: general practitioner
