# Supplementary material for: Using Mobile Phone Apps to Deliver Rural General Practitioner Services: Critical Review Using the Walkthrough Method
Source: JMIR Form Res. 2022 Jan 25;6(1):e30387. doi: 10.2196/30387 (PMC8826308; doi:10.2196/30387)
Supplement: Multimedia Appendix 1 [file formative_v6i1e30387_app1.docx]

Multimedia Appendix **1.** Data collection template related to the walkthrough method [44].

| Scan | Considerations for collecting data |
| --- | --- |
| **Environmental walkthrough** | |
| The first step is to consider the *environment* of expected use, including social, political, economic, and cultural contexts.  As part of this step, the app’s anticipated users and purposes are considered along with the business model and modes of governance.  Along with the app itself, data for this may include review of corporate materials such as websites, social media pages, policies, and media releases. | - Vision   - Consider the app’s purpose, target user base, and scenarios of use.   - What is it supposed to do? How can it be used and who is it for?   - Consider any taglines.   - What images are used?   - Are there any new observations that can be gleaned from the app store, websites, blogs, marketing, and public statements? - Operating model   - Consider the business strategy and revenue sources, which indicate underlying political and economic interests.   - Was it *Freemium* (basic access in exchange for data and more perks with payment)? Did it include in-app advertisements?   - Were there any partnerships with other platforms or multisided markets?   - Were there any channels of outside funding or donations? - Governance   - Examine how the app provider seeks to manage and regulate user activity.   - What were the guidelines, terms, and rules; hard-coded (eg, censorship of hashtags), copyright licensing, and FAQs^a^; and app store regulations?   - Was there any user mutual surveillance? |
| **Technical walkthrough** | |
| Having established the environment of expected use, the next step in the walkthrough is the technical walkthrough. This step involves engaging with the app interface and considering the mediator characteristics. Interrogating and analyzing these characteristics makes visible the features of the interface that might otherwise go unnoticed in everyday use. It draws attention to how the app seeks to configure relations among actors and to transform meaning through the interactions invoked. | - User interface arrangement   - What were the navigation options?   - What about the design and flow? - Functions and features   - What activities were entailed?   - Were there any mandatory fields? - Textual content and tone   - What text was used?   - Did it include a particular order of menus? - Symbolic representation   - What was the look and feel of the app?   - What images were used?   - What colors were applied? - Stages and steps available when using the app   - How were registration and entry enabled?   - Was there access via a website or app store? What was the process for downloading?   - Automatic functions—information imported?   - Were there permissions and location-based services?   - Was it accessible for everyday use?   - Were there functions for suspension, closure, and leaving?   - Were there any unexpected practices and user-led activities that the app promoted? - Levesque framework [48]   - Approachability: what was the app’s transparency, outreach, and information about available treatments and services?   - Acceptability: what were the professional values, norms, culture, and gender and how could they influence how the app is perceived?   - Availability and accommodation: where is the app available? What are the appointment times and mechanisms?   - Affordability: were there any indirect and direct costs of the services on offer, modes of payment, subsidies, and costs of accessing the app?   - Appropriateness: what is the appropriateness of the services offered, quality of their delivery (technical and interpersonal), and coordination and continuation of care? |

^a^FAQs: frequently asked questions.
