# Supplementary material for: Using Mobile Phone Apps to Deliver Rural General Practitioner Services: Critical Review Using the Walkthrough Method
Source: JMIR Form Res. 2022 Jan 25;6(1):e30387. doi: 10.2196/30387 (PMC8826308; doi:10.2196/30387)
Supplement: Multimedia Appendix 2 [file formative_v6i1e30387_app2.docx]

**Multimedia Appendix 2.** A summary of the app business models and allowances^a^.

| Feature | App 1 | App 2 | App 3 |
| --- | --- | --- | --- |
| Started | - 2015, free to download | - 2011, free to download | - 2018, free to download |
| Founders | - Trained pharmacist with business interests | - Trained physician with interests/skills in engineering, IT^b^, and business | - An ex-GP^c^ practice manager |
| Front of house | - No receptionist - Direct to physician - Open 24/7, 7 days a week, but main business hours 8 AM-6 PM | - No receptionist - Direct to physician - Open 7 AM-7 PM, 7 days a week, and physicians can offer appointments outside of these hours | - No receptionist - Direct to physician - Open 6 AM to midnight, 7 days a week |
| Business model | - Stand-alone MedTech company. Partners are private health firms such as Sigma Healthcare, associated pharmacy brands (such as Amcal, Guardian, and Discount Drug Store pharmacies), and Universal Music Group. | - Had a Series A capital raise in 2015 and became ASX^d^-listed. Sonic Healthcare and leading private health insurer HCF^e^ are partners and investors. Intention to be a one-stop telehealth management system tailored to usability and accessibility to attract specialist sign-up, making it easy for the GPs to source specialist telehealth consults for their patients. | - Has partnerships with pharmacies. |
| Technology requirements | - Minimum requirement is 3G capability. If it drops out, they call the user on mobile or make another appointment. | - Any type of device. Allows the user to test internet and video on their webpage and has a video for new users showing how it works. | - Any type of device. Needs a camera and speakers connected/installed on the device. |
| Accessible GPs | - Pick from male and female physicians currently on the web, approximately 3-4 on the web at any time, shows their name and picture and how many patients are waiting are and the time the user will spend in the web-based queue before they book a consult if they want an appointment now, or they can choose a time if they want a consult later. Can set appointments in different languages (but not always available). | Pick from a selection of physicians (potentially 2-4), male and female, and the user can choose times for an appointment. GPs listed by name and location. When the user books and pays, it sends them to a waiting room. | - Unable to see GP options to book with as we did not enter payment details. Possible that they are allocated based on *next available*. No names or pictures. |
| GP payment | - Physicians will be paid fees on a monthly basis for hours worked or as a percentage of fees, with difference between in-hours and after-hours services. | - It is not disclosed how physicians are paid. It notes physicians set their own consultation fees. | - It is not disclosed how physicians are paid. |
| Allied services | - Yes, psychologists, 24/7 access to pharmacy, dieticians, and physiotherapists, but not all allied health services are available when trying to book. | - Yes, a sister site to see related therapists. Also offered on this site: social workers, endocrinologists, psychiatrists, and psychologists. | - Only by referral, not built-in. |
| Time | - All consults ≤15 min, not shown until booking made. | - All consults ≤15 min, not shown until pressing *book*. | - Not shown on the app. Website shows consults charged by duration—brief (<10 min), standard (>10 min), long (>20 min), and prolonged (>40 min), automatically timed. |
| Cost | - Not shown on the app but on the website. Differentiated costs by service type, from Aus $60 (US $43.37) for a physician, from Aus $27.95 (US $20.21) for a script, and from Aus $60 (US $43.37) for a medical certificate. Prescription handling fee (7 days a week) is Aus $2.95 (US $2.13). After hours, appointments are Aus $90 (US $65.06). Enter payment details to proceed to consult. | - Shown when pressing *book*, Aus $40 (US $28.92) or Aus $50 (US $36.15) deducted as varying by 10- or 15-minute time slots at discretion of the GPs on the web. Enter payment details to proceed to consult. | - On the app under FAQs^f^, which can be read before booking an appointment, by link. The website also shows billing per duration (Aus $35-$105 [US $25.30-$75.91]). Enter payment details to proceed to consult. Payment happens at conclusion of web-based consultation (automatically deducted based on time). Must consent to pay fee when requesting a consult. |
| Payment method | - Requires credit card, but the user cannot see this until they register and book. | - Options of credit card, PayPal, and Direct Deposit. | - Requires credit card, but the user cannot see this until they register and book. |
| Rebates for medical care or concessions | - Medicare rebates not available. No concessions. | - Only Medicare rebates for telehealth for specialist appointments, and it allows the user to see if they are in an eligible area (rural). No concessions. | - Medicare bulk billing for patients aged <12 months, homeless, or living in a COVID-19 hot spot; time-limited. Upon requesting a consult, usermakes a declaration for this. No concessions. |
| Refund | - Can cancel appointment but no refund possible. Physicians may cancel appointments at their discretion if they think the user is not eligible for the service and will issue a refund. | - Can cancel appointment with 24 hours’ notice (no fee incurred) if the user emails them. | - Payment released if consult request is not successful or cancelled. |
| Rural marketing | - None | - Helps those “possibly thousands of kilometres away.” - “...deliver care from anywhere in Australia to patients in metro, regional, remote and foreign locations.” | - Useful for “...people who need the access as their town either does not have a GP, or if it does, they may be booked out for 3 or 4 weeks...” |

^a^Environmental and technical walkthrough notes combined for easy reference.

^b^IT: information technology.

^c^GP: general practitioner.

^d^ASX: Australian Stock Exchange.

^e^HCF: A leading private health insurer

^f^FAQs: frequently asked questions.
