# Supplementary material for: Using Mobile Phone Apps to Deliver Rural General Practitioner Services: Critical Review Using the Walkthrough Method
Source: JMIR Form Res. 2022 Jan 25;6(1):e30387. doi: 10.2196/30387 (PMC8826308; doi:10.2196/30387)
Supplement: Multimedia Appendix 3 [file formative_v6i1e30387_app3.docx]

**Multimedia Appendix 3.** A summary of the app appointment functions and utility^a^.

| Feature | App 1 | App 2 | App 3 |
| --- | --- | --- | --- |
| Claim of market | - 70% of GP^b^ presentations can be handled by telehealth (based on American research that is not cited). | - 80% of normal GP services can be done on the web. | - None |
| Booking clinical services | - First page denotes the following: “doctors online when you need them.” - Goes straight to a picture of a female physician with a stethoscope where the user can choose the following options from a list: see a physician, order a prescription, obtain a medical certificate, and see another health expert. - Script renewal without seeing a physician (medical review after filling in a questionnaire). - Can book consult now or later. - When choosing a physician from those on the web, the user needs to complete the following questions: reason for appointment, pharmacy selection, and general medical questions (smoking, alcohol, cardiovascular, allergies, current medications, disorders, major surgeries, height, weight, and blood pressure), but these are open text fields. Requires consent and read and understood T&C^c^ and privacy policy. - Also proposes corporate wellness packages (seeing employees for occupational health when they are at work). | - First page goes straight to a date and drop-down menu where the user can pick GP, endocrinology, psychiatry, psychology, or social work and click on the main green button (“Book appointment”), which leads them straight to physician booking for now or later. - Website and “learn more” button on the app propose they provide prescriptions, referrals, medical certificates, mental and sexual health services, and support for other conditions. They allow immediate prescriptions (faxed to the nearest chemist delivery service or home delivered) “from the comfort and convenience of home...office.” - The website proposes they address weight loss support, alcohol and drug support, infectious disease prescriptions (sensitive issues such as STIs^d^), and workers’ compensation certificates. - Allows storing, forwarding, and appointment reminders, among other things. - On the app, the user can choose black buttons (watch video, learn more, register now, contact us, and QR code). | - First page denotes the following: “access to physicians from your mobile phone.” Leads to page that shows average wait time and the following statement—“doctors online now”—where the user can pick “inbox” or “request a consult.” - Only for booking a consult immediately, not later. The app has a link to FAQs^e^, including “what can I use instant consult for.” - When booking, the user is asked to list Medicare eligibility and consent. Then, they get to the list “nature of consult,” where options are “medical certificate, prescription, referral, pathology request, radiology request and other.” - Not clear what “other” entails. For any requested consult, there is a page to provide additional information and upload documentation before the consultation proceeds. - The consult proceeds if the user types any free text in but will not unless the text field is populated. The user is then told not to proceed if it is an emergency and asked to “confirm” before booking. |
| Disclaimer | - Does not propose to cover emergency or particular medications. Emergencies not defined. - Physicians reserve the right not to treat the user if the user is complex. - Does not state what should be done by the normal GP face-to-face, except that the schedule 4 drugs that have the potential to cause harm should be sourced from the user’s regular GP. - They do not represent, warrant, or guarantee the quality of any medical advice provided by a physician during a consultation or otherwise. No mention of service backup (eg, chance for GP to receive coconsultation support for difficult cases). | - Rules minimal things out, except some drugs, and emergencies. Emergencies are not defined. - Under “disclaimer,” it says the following: “If you have a medical emergency please stop now and contact local emergency services.” Under “disclaimer,” it says the following: “If you are in Australia dial 000. If you are overseas, and don’t know who to call, we recommend you contact the Australian Government’s 24-hour Consular Emergency Centre on +61 2 6261 3305.” - Does not state what should be done by the normal GP face-to-face. - However, it says the following in disclaimers: “Circumstances beyond our control may render it impossible to offer you an adequate service in which case you should seek the services of a local doctor.” - Individual physicians have services that they will not provide, which are visible when a consult is requested, such as “unable to process mental healthcare plans.” No mention of service backup (eg, chance for GP to receive coconsultation support for difficult cases). | - Does not propose to do complex or emergency things (emergency is defined on the website Q&A^f^ as the following examples: “chest pain, head or spinal injuries, severe bleeding, loss of movement, breathing difficulties and reduced level of consciousness”). - All services carry the following term: “[We will do this]...when clinically appropriate.” - Once a service is requested (eg, prescription), the things that cannot be issued become clear (eg, Panadeine Forte). - They also note the following: “We rarely issue a Medical Certificate covering more than 3 days. The details issued on the Medical Certificate is up to the physician and what they deem suitable during consultation.” - “If you require a ‘Fit for Work’ Certificate, Centrelink Certificate, University Form completed, Work Cover or Gym Cancellation Certificate—you will need to present to a GP in-person to undergo a physical examination.” - Mental health service plans also cannot be generated. - They also note that, as they are trying to balance supply with demand, the user “may join a queue until the next available doctor connects to you.” - No mention of service backup (eg, chance for GP to receive coconsultation support for difficult cases). |
| Use of service from overseas | - No | - Yes | - No |

^a^Environmental and technical walkthrough notes combined for easy reference.

^b^GP: general practitioner.

^c^T&C: terms and conditions.

^d^STIs: sexually transmitted infections.

^e^FAQs: frequently asked questions.

^f^Q&A: questions and answers.
