# Supplementary material for: Using Mobile Phone Apps to Deliver Rural General Practitioner Services: Critical Review Using the Walkthrough Method
Source: JMIR Form Res. 2022 Jan 25;6(1):e30387. doi: 10.2196/30387 (PMC8826308; doi:10.2196/30387)
Supplement: Multimedia Appendix 4 [file formative_v6i1e30387_app4.docx]

**Multimedia Appendix 4.** A summary of the app quality, privacy, and troubleshooting^a^.

| Feature | App 1 | App 2 | App 3 |
| --- | --- | --- | --- |
| GP^b^ qualifications | - Shown under the name of the GP booked. Shows title of qualifications and country where qualifications were attained but no evidence of the institution from which the qualifications were obtained or the date. | - Shown once the user selects a GP to book, with title of qualifications; university, specialty college, and year of attainment; and special interests. | - Cannot see GPs nor qualifications until payment details are entered. |
| How GPs are employed | - Subcontracted service providers with their own ABN^c^ and liable for their own PAYG^d^, workers’ compensation, and superannuation. | - There is a phone number for GPs to sign up to provide services; their screening for eligibility is not visible. Not clear how GPs are employed. | - Proposes to allow GPs to work from the comfort of their own home. Has screening questions so physicians can determine eligibility: registered fellow of GP college and no costs. Not clear how GPs are employed. |
| Privacy | - Privacy policy. They do not collect, have access to, or store intimate details of the users’ consultations or confidential medical records, but medical records are recorded and kept by the user’s consulting physician. Allows patients to access their own record. Consent needed to share personal data with third parties. - Not clear how to delete the user’s profile. | - Privacy policy. They do not send the user’s information or the outcome of the consultation to other physicians unless requested to do so by the user. They use security suppliers who also support banks. - It notes that they are required by law to keep the user’s data for auditing. - Not clear how to delete the user’s profile. | - Privacy policy, but hard to read via the app as the screen cannot be enlarged. Encrypted software is outlined, trusted by >99.9% of current internet users. There is minimal information about managing medical records and privacy. Required by law to keep documentation such as profile for auditing. - Not clear how to delete the user’s profile. |
| Safety and quality | - Implied based on getting to see a physician (of the choices available on the web) quickly and for a specific purpose. Also implied by “using the latest in web technology,” training and onboarding physicians, and using Best Practice Software integrated with their portal. Use registered physicians not necessarily GP-qualified. | - They use the same formal accreditation processes used by major private hospitals to validate the credentials of their physicians before they are available for appointments. A contact point is provided if the users have a concern. Use Australian-registered, fully qualified and experienced physicians. | - Screening questions for physicians wanting to join the service and a physician relationship manager (contactable at all times) to promote connection to the physicians involved. Use Australian-registered, fully qualified and experienced physicians. |
| Children | - Generic. Children not mentioned. No proof of age required during sign-up other than entering DOB^e^, mobile phone number, and credit card. | - Generic. Children not mentioned. No proof of age required during sign-up other than entering DOB, mobile phone number, and possibly credit card. | - Consultations available for children if the parent makes the child a profile. No mention of proof of parenthood. Must be aged ≥17 years to use the service. If aged <17 years, the user must be in the presence of an adult at all times and have the consent of a parent or guardian. However, no proof of age is required during sign-up other than entering DOB, mobile phone number, and credit card. |
| Ongoing care | - No mention of how health care will be followed up or coordinated. If using a quick script service, the regular GP’s details are provided, and the regular GP registers with their service, a quick script request can be sent directly to them. | - No mention of how health care will be followed up or coordinated. Allows patients to see their own physician if their physician registers with the service. - Their IT^f^ platform is established to accommodate multiple GPs and specialists and make it easy for GPs to find specialists happy to provide telehealth consults. | - No mention of how health care will be followed up or coordinated. |
| FAQs^g^ and contacting them | - Has an FAQ page on the website. - Deep into the webpage, there is an Australian landline number or email to contact if the user has a question. | - Has FAQs. - Has a 1300 number to contact to get in touch with the service and shows office locations in 2 states. | - Has FAQs available. - Has 1300 number contact details for talking to support team by email or phone. |
| Teaching | - No evidence of teaching | - No evidence of teaching | - No evidence of teaching |

^a^Environmental and technical walkthrough notes combined for easy reference.

^b^GP: general practitioner.

^c^ABN: Australian Business Number.

^d^PAYG: pay as you go.

^e^DOB: date of birth.

^f^IT: information technology.

^g^FAQs: frequently asked questions.
